# The impact of maternal depressive symptoms and traumatic events on early childhood mental health in conflict-affected Timor-Leste

**DOI:** 10.1192/bjo.2022.20

**Published:** 2022-02-24

**Authors:** Susan J. Rees, Mohammed Mohsin, Louis Klein, Zachary Steel, Wietse Tol, Mark Dadds, Valsamma Eapen, Zelia da Costa, Elisa Savio, Natalino Tam, Derrick Silove

**Affiliations:** School of Psychiatry, Faculty of Medicine, University of New South Wales, Australia; School of Psychiatry, Faculty of Medicine, University of New South Wales, Australia; and Mental Health Research Unit, Liverpool Hospital, New South Wales Health, Australia; School of Psychiatry, Faculty of Medicine, University of New South Wales, Australia; and Mental Health Research Unit, Liverpool Hospital, New South Wales Health, Australia; School of Psychiatry, Faculty of Medicine, University of New South Wales, Australia; Department of Mental Health, Centre for Global Health, Johns Hopkins University, USA; School of Psychology, Faculty of Science, University of Sydney, Australia; School of Psychiatry, Faculty of Medicine, University of New South Wales, Australia; and Academic Mental Health Unit, Liverpool Hospital, New South Wales Health, Australia; School of Psychiatry, Faculty of Medicine, University of New South Wales, Australia; School of Psychiatry, Faculty of Medicine, University of New South Wales, Australia; School of Psychiatry, Faculty of Medicine, University of New South Wales, Australia; Brain Sciences, University of New South Wales, Australia

**Keywords:** Conflict-affected populations, maternal depression, psychological trauma, child mental health

## Abstract

**Background:**

Longitudinal studies are needed to examine the association between maternal depression, trauma and childhood mental health in conflict-affected settings.

**Aims:**

To examine maternal depressive symptoms, trauma-related adversities and child mental health by using a longitudinal path model in conflict-affected Timor-Leste.

**Method:**

Women were recruited in pregnancy. At wave 1, 1672 of 1740 eligible women were interviewed (96% response rate). The final sample comprised 1118 women with complete data at all three time points. Women were followed up when the index child was aged 18 months (wave 2) and 36 months (wave 3). Measures included the Edinburgh Postnatal Depression Scale, lifetime traumatic events and the Child Behaviour Checklist. A longitudinal path analysis examined associations cross-sectionally and in a cross-lagged manner across time.

**Results:**

Maternal depressive symptom score was associated with child mental health (cross-sectional association at wave 2, *β* = 0.35, *P* < 0.001; cross-sectional association at wave 3, *β* = 0.33, *P* < 0.001). The maternal depressive symptom score at wave 1 was associated with child mental health at wave 2 (*β* = 0.12, *P* < 0.001), and the maternal depressive symptom score at wave 2 showed an indirect association with child mental health at wave 3 (indirect standardised coefficient 0.23, *P* < 0.001). There was a time-lagged relationship between child mental health at wave 2 and maternal depression at wave 3 (*β* = 0.08, *P* = 0.02).

**Conclusions:**

Maternal depressive symptoms are longitudinally associated with child mental health, and traumatic events play a role. Maternal depression symptoms are also affected by child mental health. Findings suggest the need for skilled assessment for depression, trauma-informed maternity care and parenting support in a post-conflict country such as Timor-Leste.

Evidence suggests that depression in maternal caregivers is associated with child behaviour and developmental problems.^1,2^ Depression is a common mental health problem affecting women in the childbearing years, and studies suggest that the prevalence is higher in the antenatal period (period from the start of the pregnancy to the onset of labour).^3,4^ Globally, antenatal depression is estimated to range between 15 and 65%, with much higher rates in low- and middle-income countries.^4^ Maternal depression can also negatively affect the child, with studies in general populations, including low-and-medium-income countries, consistently showing the adverse effects of maternal depression on child mental health and behavioural outcomes.^5,6^ Impaired parenting and bonding have been proposed as possible mechanisms leading from maternal depression to child mental health problems.^1,2,6^ With impaired parenting, children may be exposed to dysphoric mood, irritability, confusion, helplessness and hopelessness in mothers with depression.^7^ Further, the psychological unavailability of mothers experiencing depression can interfere with attachment and bonding with the child. Mothers with depression may also be less consistent or considered with discipline, and they may also be role models of unwanted behavioural responses, reactions and interactions.^7^ The effects of maternal depression on child mental health can be identified in early childhood, most often studied as internalising and externalising forms of psychological symptoms.^6^

## Factors associated with maternal depression and child mental health

Maternal depression and associated child mental health problems can also be accounted for by shared genetic vulnerabilities, epigenetic, social and economic factors.^8,9^ It is further widely appreciated that genetic and prenatal factors are not able to fully account for the psychological difficulties found in children of caregivers with depression.^10,11^ In fact, research tends to support a view that socially constructed, contextual factors are best to identify and respond to the trajectory and effects of maternal depression on child mental health.^12^

Exposure to trauma may also contribute to maternal and child mental health problems.^13^ Women of childbearing age in Timor-Leste have experienced conflict-related traumas, such as sexual abuse and other human rights violations, during the Indonesian occupation, as well as internal displacement during the 2006 and 2007 periods of civil conflict. These experiences are consistent with the global experience of many refugee and conflict-affected women.^14,15^ The adverse effects of conflict trauma on refugee women's mental health has also been demonstrated among Timorese women.^14,16,17^

## Trauma and conflict in maternal depression

Maternal depression and post-traumatic stress symptoms predictors of mental health problems in children up to 6 years of age.^18^ Longitudinal studies from conflict-affected Afghanistan, Pakistan,^19^ and Sierra Leone,^20^ have reported intergenerational mental health effects in children. Trauma associated with war and conflict can disrupt the relationship between mother and child, altering how the caregiver manages compounding daily social, family and parenting challenges in low socioeconomic environments.^14^ Furthermore, trauma and related stress can affect how the caregiver experiences their attachment to the child, their confidence as a parent and their capacity to identify and respond to the child's needs.^21^

The predominance of a trauma-focused model has given rise to an emphasis on post-traumatic stress symptoms in studies of maternal mental health in conflict-affected countries.^13,22^ Our research and other studies conversely show that the prevalence of depression in conflict-affected women is equally if not more important, particularly during the antenatal period.^3,4,23^ Exposure to trauma may increase women's risk for depression, both in general populations and conflict-affected groups.^24^ Although research has shown a compelling association between caregiver and child mental health in conflict settings, none has focused longitudinally on depression and its relationship with maternal trauma and child mental health.

## Need for longitudinal evidence

Most cross-sectional studies have assumed directionality in the relationship between maternal mental health problems and future child development or psychological issues, usually via the mechanism of impaired parenting. Few studies have undertaken longitudinal studies to deliberately examine if child mental health could also have a negative effect on maternal mental health.^25^ Challenging or concerning child behaviours (e.g. being excessively withdrawn or overtly aggressive or defiant) can theoretically trigger or exacerbate maternal stress and mental health problems; an effect demonstrated in studies with caregivers of autistic children and those with disabilities.^26,27^ Elgar et al also reported that mothers of children with adjustment problems are regularly exposed to and potentially psychologically affected by aggressive, hyperactive, delinquent or emotionally disturbed children.^7^ This gap in the conflict and refugee literature is remarkable, given that associations between other types of stressful conditions, such as economic and social adversity, on refugee women's mental health have been demonstrated.^13,20^ Only longitudinal studies can examine directionality in these factors and their inter-relationships.^28^

## Timor-Leste

Timor-Leste is a low-income country exposed to prolonged conflict, and has experienced significant conflict-related periods of violence. During the resistance period, a war was waged against the brutal Indonesian occupation (1975–1999). Timorese men suffered extensive human rights violations, including torture and arbitrary detention, and women were subjected to rape, forced marriages and coercive removal of their children.^14,16,29,30^ Family disruptions caused by death and displacement were extensive, leaving many women with no or limited support. After an interregnum of peace surrounding national independence (2002), Timor-Leste experienced a period of internal conflict (2006–2007) resulting in further violence, deaths and destruction of property. In addition, rates of domestic violence are high, with men's exposure to human rights trauma contributing to the problem, along with gender inequalities.^14,16,29,30^ It is one of the poorest countries in the world, and women currently carry the burden of providing and caring for children, a challenge that is exacerbated by lack of control over reproduction and absence of spousal support.^14,16,29,30^ In addition to this burden on families is the issue of prevailing poverty, strongly associated with mental disorder and poor functioning.^14,16,29,30^

This study sought to address a gap in knowledge by examining maternal depressive symptoms, trauma-related adversities and child mental health by using a longitudinal path model based on data collected in conflict-affected Timor-Leste. Previously, we reported cross-sectional evidence from the same sample that showed robust associations between conflict-related trauma and depressive symptoms among pregnant women.^16,29^ We have also shown qualitatively that exposure to conflict-related trauma and poverty may affect maternal mental health and parenting of young children.^31^ We therefore hypothesised that trauma and depressive symptoms would lead to childhood behavioural problems in the offspring of the women in our cohort study studied sequentially. We also hypothesised that there would be a relationship between child mental health and maternal depression.

We collected data every 2 years at three time points, using the Edinburgh Postnatal Depression Scale (EPDS) to measure depressive symptoms in women. The measure was rigorously tested in this setting in a previous study (the process is described below).^16^ The Child Behaviour Checklist for ages 1.5–5 years (CBCL/1.5–5) was used to measure psychological problems in their children.^31^ Given our general focus on the study of relationships between theoretically supported variables associated with maternal depression, traumatic events and child mental health, we considered the overall CBCL score rather than examine the subgroups of internalising and externalising problems. We included age of caregiver, child gender, past conflict-related traumatic events, and EPDS and CBCL scores in our statistical model.

## Method

### Study design and participants

The DILI (*Desenvolvimentu Isin-d'iak Labarik no Inan*; literal translation: ‘Development and Well-being of Children and Caregiver’) study interviewed women in three waves over the period 2013 to 2018.^32^ Women were recruited consecutively from all four large public antenatal clinics in the Dili District of Timor-Leste, which includes the capital city. The clinics provide services to >90% of pregnant women in the district, which is home to 20% of the country's 1.1 million persons.^33^ Participants were recruited in the second trimester (3–6 months) of pregnancy, the peak period of registration at clinics. Women were followed up at home when the index child was aged 18 months (wave 2) and 36 months (wave 3). Excluded were women with psychosis, profound intellectual impairment or severe medical illnesses. Details of recruitment and retention are presented in the flow chart in Supplementary File 1 available at https://doi.org/10.1192/bjo.2022.20.

At wave 1, we interviewed 1672 of 1740 eligible women. Of these, 1303 (78%) were retained at wave 2 and 1140 were retained at wave 3 (68.2% of the wave 1 sample) (see Supplementary File 1 for details). Most non-participants could not be located or contacted after five attempts, and a minority had moved to distant parts of Timor-Leste or refused to participate. The final analytic sample comprised 1118 women (67% of wave 1 sample) with complete data at all three time points.^16^

### Survey measures

Measures were repeated at all waves and the CBCL was completed by maternal caregivers in relation to the index child at waves 2 and 3.^34,35^ All measures had been subject to extensive cultural and linguistic adaptation, including translation and back-translation into the *lingua franca*, Tetum.^36^

#### Sociodemographic characteristics

We included relevant items from the Timorese national census, including usual place of residence, age, marital status, highest level of education and employment status.

#### Maternal depressive symptoms

We used a culturally adapted and locally piloted version of the widely used EPDS, comprising ten items scored on a four-point self-rated Likert scale (0–3), generating a summary score ranging from 0 to 30; higher scores indicate more severe depressive symptoms.^37^ Internal reliability (Cronbach's alpha) for the EPDS at waves 1, 2 and 3 were 0.70, 0.79 and 0.80, respectively. Although the EPDS is not a diagnostic measure, a total symptom score of ≥13 is considered to indicate a clinically significant level of depression.^37^ For comparison purposes, we provide prevalence rates based on this predetermined cut-off. However, in the path analysis, we used the total depressive symptom score.

#### Conflict-related traumatic events

We previously adapted and tested the 23 items for lifetime traumatic events of the Harvard Trauma Questionnaire for Timor-Leste.^37^ Items included political imprisonment, assault, torture, witnessing murder, exposure to atrocities, traumatic losses/separations of family or close others, and deprivation of medical care for self or others in situations of severe illness. Consistent with past research in the field, we generated a total trauma count by adding those events that had occurred at least once in the person's lifetime (scored 1).^38,39^ We applied a conventional scoring approach by generating a summary of lifetime traumas (each item rated as occurred (1) or absent (0)). Our past experience working in the country indicated that attempts to ascertain the occurrence or enumerate the instances of exposure to the same trauma generated inaccurate findings, consistent with a society with low literacy and numeracy levels and little focus on recording dates and times. We applied the traumatic events count at wave 1, given that no episodes of mass conflict had occurred subsequently.

#### CBCL/1.5–5

The CBCL/1.5–5 was used to assess mental health (specifically behavioural/emotional problems and competencies) in the index child for ages 1.5–5 years.^34^ The CBCL is an empirically derived measure that is completed by the child's parent or caregiver. The CBCL has major advantages, including good psychometric properties and national norms based on thousands of children. Standardised scores permit comparisons between gender and across age groups and cultures, although raw scores have been recommended for research.

Caregivers rated the 99 CBCL descriptors on a three-point Likert scale (0, not true; 1, somewhat true; 2, certainly true). Although several methods exist for analysing the data, we selected the total CBCL/1.5–5 problem score for each wave (calculated by adding all 99 items, with a possible range of 0–297). The method for analysis for total score was selected because of the broad focus of the study.^40^ The internal reliability of the 99 items based on Cronbach's alpha (*α*) at waves 2 and 3 were high, at 0.92 and 0.91, respectively, consistent with previous studies in other settings and cultures.^35^

### Field team

The field team comprised 18 Timorese women who received 2 weeks of intensive training, and ongoing supervision in field work to ensure all safety and ethical protocols were applied. They were supervised by the Australian team applying the battery of measures among women in the relevant clinics. All field team members were required to achieve 100% interrater reliability with supervisors when testing the use of the EPDS before commencing the main study. All interviews were conducted in Tetum, which is the *lingua franca* of Timor-Leste. Further supervision was provided throughout the course of the study, as well as regular feedback and ongoing in-service training to maintain the quality of assessments, data recording and entry.

### Ethics

The study was approved by the Human Research Ethics Committee of the University of New South Wales and the Ministry of Health of Timor-Leste (approval number HC180697). Signed or witnessed verbal consent was obtained from all participants, and interviews were conducted under conditions of strict privacy.

### Statistical analyses

We document the descriptive data for all indices used in the present analysis. The mean and s.d. for the total CBCL score are presented according to the child's gender, caregiver's age and maternal EPDS threshold score (≥13).^39^ To examine the significant differences between and across groups, we applied the *t*- and *F-*test statistics for bivariate analyses (*P* < 0.05). Theoretically relevant indices that showed a bivariate relationship with either maternal depressive symptoms or the CBCL score or both, were entered into the path model within a structural equation modelling framework.^33^ These indices included gender of the index child, age of caregiver, conflict-related traumatic events, maternal depression score and child behaviour index. The structural equation model was designed to test the following associations: (a) inter-relationships of maternal depressive symptoms across the three waves; (b) inter-relationships of the CBCL total score at wave 2 and wave 3; (c) cross-sectional and time-lagged associations between maternal depressive symptoms and the CBCL; (d) paths leading from conflict-related traumatic events to maternal depressive symptoms at relevant waves; (e) paths leading from traumatic events to the CBCL at waves 2 and 3; and (f) influence of caregiver's age and child's gender on the CBCL at waves 2 and wave 3.

Conventional statistics assessed model fit including a non-significant chi-squared test. We further applied other estimation techniques to ensure model fit, including comparative fit index (CFI) >0.90, Tucker–Lewis Index (TLI) >0.90, root-mean-square error of approximation (RMSEA) <0.08 and standardised root-mean-square residual (SRMR) <0.08. The analyses were performed in SPSS for Windows version 25 and MPLUS (Muthen & Muthen; see https://mplus.software.informer.com) version 7.1.

## Results

### Descriptive data and bivariate analyses

The mean age increased from 26.3 to 29.9 years, i.e. about a 3.6-year or 43-month increase (which is >36 months). As the age increased, a significant number of women who were under 25 years at baseline (wave 1) were moved to older age groups, and because of this, in waves 2 and 3, the number of women in the older groups increased (and therefore decreased in younger age groups). Marital status, education and employment status showed little change over time (Table 1). Of the 1118 children, 516 (46.2%) were girls and 602 (53.8%) were boys.

**Table 1:** Sociodemographic characteristics, conflict-related traumatic events, maternal caregiver's depressive symptoms and child mental health measures

	Wave 1 survey (*n* = 1118)		Wave 2 survey (*n* = 1118)		Wave 3 survey (*n* = 1118)	
Sociodemographic characteristics and mental health indices	Number of women	Column %	Number of women	Column %	Number of women	Column %
Age group (in years)						
<25 years	445	39.8	268	24.0	166	14.8
25–29	397	35.5	419	37.5	417	37.3
≥30	276	24.7	431	38.5	535	47.9
Mean age (s.d.) [range]	26.3 (5.1)	[15−44]	28.4 (5.3)		29.9 (5.2)	[18−50]
Marital status						
Married	302	27.0	Not collected		Not collected	
Engaged/living together	813	72.7				
Separated/divorced	3	0.3				
Still married/living with same person: yes	−		1089	97.4	1077	96.3
Highest level of educational attainment						
None or primary school	164	14.7	183	16.4	Not collected	
Junior/senior high school	663	59.3	656	58.7		
Technical college/diploma	72	6.4	67	6.0		
University	219	19.6	212	18.9		
Employment status						
Unemployed	732	65.5	757	67.7	Not collected	
Paid employment/small trade/farming	386	34.5	361	32.3		
Conflict-related traumatic events						
Total traumatic events, mean (s.d.)	5.3 (3.4)		3.7 (2.4)		1.2 (1.4)	
At least one traumatic event	1082	96.8	1066	95.4	681	60.9
Edinburgh Postnatal Depression Scale score ≥13.0	210	18.8	132	11.8	91	8.1
Total score, mean (s.d.)	8.5 (4.3)		6.4 (4.8)		5.0 (4.5)	
Child's gender						
Girl			516	46.2	516	46.2
Boy			602	53.8	602	53.8
Total Child Behavior Checklist score, mean (s.d.)			48.1 (19.4)	[0−135]	40.1 (16.6)	[0−97]

The number of women exceeding the EPDS threshold for depressive symptoms declined in a stepwise manner: from 210 of 1118 (18.8%) at wave 1 to 132 of 1118 (11.8%) at wave 2 and 91 of 1118 (8.1%) at wave 3 (McNemar's test, *P* < 0.001). The mean CBCL score for boys was higher than that for girls at both wave 2 (boys: mean 49.3, s.d. 19.7; girls: mean 46.7, s.d. 18.8; *P* = 0.03) and wave 3 (boys: mean 40.9, s.d. 16.6; girls: mean 39.0, s.d. 16.7; *P* = 0.05), although absolute differences were small (Table 2). The maternal depressive symptom score at wave 1 was correlated with the same index at wave 2 (*r* = 0.27, *P* < 0.001) and wave 3 (*r* = 0.24, *P* < 0.001); and the maternal depressive symptom score at waves 2 and 3 were also significantly correlated (*r* = 0.34, *P* < 0.001). The child behaviour index score declined from 48.1 (s.d. 19.4) at wave 2 to 40.1 (s.d. 16.6) at wave 3 (paired *t*-test, *P* < 0.001), although the serial scores remained statistically correlated (*r* = 0.46, *P* < 0.001).

**Table 2:** Mean of total Child Behaviour Checklist problems score (waves 2 and 3) by maternal caregiver age, child's gender and maternal depressive symptoms threshold category (waves 1–3)

	Number of women	Total CBCL problems score	
		Wave 2	Wave 3
		Mean (s.d.)	Mean (s.d.)
All	1118	48.1 (19.4)	40.1 (16.6)
Maternal caregiver age (in years)			
<25	445	50.3 (19.9)	41.8 (17.3)
25–29	397	47.5 (19.8)	39.8 (16.6)
≥30	276	45.4 (17.5)	37.7 (15.4)
*F*-test: *P*-values		*P* = 0.003	*P* = 0.004
Depressive symptoms, wave 1			
Not depressed (EPDS score <13)	908	46.5 (18.8)	39.1 (16.4)
Depressed (EPDS score ≥13)	210	55.1 (20.4)	44.1 (17.3)
*t*-test: *P*-values		*P* < 0.001	*P* < 0.001
Depressive symptoms, wave 2			
Not depressed (EPDS score <13)	986	46.4 (18.5)	39.1 (16.3)
Depressed (EPDS score ≥13)	132	60.7 (21.0)	47.5 (17.5)
*t*-test: *P-*values		*P* < 0.001	*P* < 0.001
Depressive symptoms, wave 3			
Not depressed (EPDS score <13)	1027	47.4 (19.1)	39.1 (16.3)
Depressed (EPDS score ≥13)	91	56.1 (21.2)	50.7 (17.1)
*t*-test: *P*-values		*P* < 0.001	*P* < 0.001
Child's gender			
Girl	516	46.7 (18.9)	39.0 (16.7)
Boy	602	49.3 (19.7)	40.9 (16.6)
*t*-test: *P*-values		*P* = 0.027	*P* = 0.049

CBCL, Child Behavior Checklist for ages 1.5–5 years; EPDS, Edinburgh Postnatal Depression Scale.

Younger age of maternal caregivers was associated with higher CBCL scores at both waves 2 and 3 (both comparisons, *P* < 0.01) (Table 2). At each relevant time point, the CBCL score for children of maternal caregivers exceeding the EPDS depressive symptom score threshold was higher than for those maternal caregivers whose EPDS score was below the depression threshold: for wave 2, mean CBCL score was 46.4 (s.d. 18.5) where maternal EPDS score was above threshold, and mean CBCL score was 60.7 (s.d. 21.0) where the EPDS score was below threshold level (*P* < 0.001); for wave 3, the corresponding CBCL scores were 39.1 (s.d. 16.3) and 50.7 (s.d. 17.1), respectively (*P* < 0.001) (Table 2).

Traumatic events occurring during past conflict measured at wave 1 were significantly associated with maternal depressive scores at all three waves (wave 1: *r* = 0.26, *P* < 0.001; wave 2: *r* = 0.16, *P* < 0.001; wave 3: *r* = 0.13, *P* < 0.001). Similarly, traumatic events were associated with the CBCL total score at waves 2 (*r* = 0.10, *P* < 0.001) and 3 (*r* = 0.17, *P* < 0.001).

### Path analysis

**Fig. 1:**
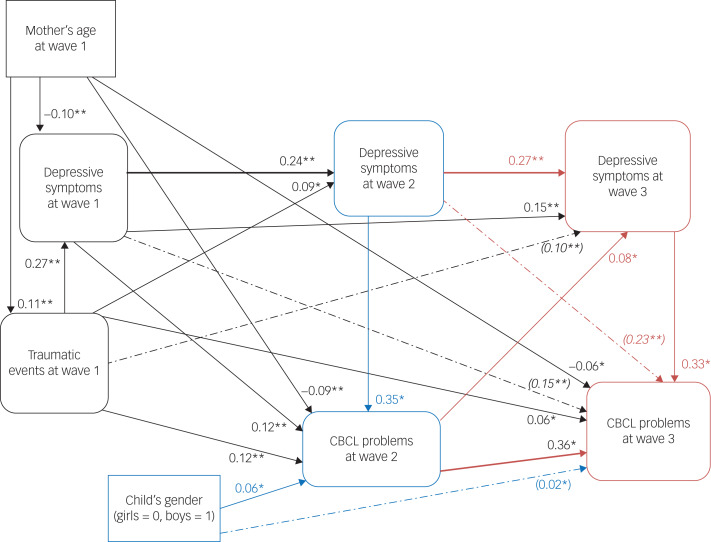
Path diagram: results from the structural equation model, with standardised direct and indirect coefficients of child's gender, mother's age and total conflict-related trauma events associated with depressive symptoms and Child Behavior Checklist (CBCL) problems at wave 2 and wave 3. The black box indicates the wave 1 measures; the black line indicates the pathways (or correlation) from the wave 1 measures to wave 1, wave 2 and wave 3 measures. The blue box indicates the wave 2 measures; the blue line indicates the correlation within wave 2 measures. The red box indicates the wave 3 measures; red line indicates the pathways (or correlation) from wave 2 to wave 2 and wave 3 measures; the red line also indicates correlation between wave 3 measures. Dashed lines show significant indirect paths. Indirect standardised coefficients are presented in bracket as italics. *P < 0.05, **P < 0.01.

Figure 1 displays the path diagram, with standardised estimates (*β*) indicating significant associations. The model achieved a good fit, with a non-significant *χ*^2^-value (*χ*^2^(9) = 9.60, *P* = 0.38), CFI = 0.99, TLI = 0.99, RMSEA < 0.001 and SRMR = 0.01.

#### Sociodemographic characteristics

Older mothers had higher exposure to conflict-related traumatic events (*β* = 0.11, *P* < 0.001). Children of younger caregivers reported higher CBCL scores at both waves 2 (*β* = −0.09, *P* < 0.01) and 3 (*β* = −0.06, *P* = 0.03) (Fig. 1 and Table 1). CBCL scores were higher for boys than girls at waves 2 (*β* = 0.06, *P* = 0.04) (a direct effect) and 3 (indirect standardised coefficient 0.02, *P* = 0.04).

#### Associations across waves between maternal depressive symptoms and child mental health scores

The wave 1 maternal depressive score was associated with the same index at waves 2 (*β* = 0.24, *P* < 0.001) and 3 (*β* = 0.15, *P* < 0.001), respectively. Similarly, the wave 2 maternal depressive score was significantly associated with the wave 3 maternal depressive score (*β* = 0.27, *P* < 0.001) (Fig. 1 and Table 2). There were association of maternal depression with child mental health at waves 2 and 3 (*β* = 0.36, *P* < 0.001) (Fig. 1 and Table 3).

**Table 3:** Structural equation model: standardised direct and indirect effects of child's gender, maternal caregiver age and total conflict-related trauma events leading to depressive symptoms and total Child Behavior Checklist problems score at waves 1 and 2

	Depressive symptoms wave 1		Depressive symptoms wave 2		CBCL problems at wave 2		Depressive symptoms at wave 3		CBCL problems at wave 3	
Candidate variables	*β*	*P*–values	*β*	*P*–values	*β*	*P*–values	*β*	*P*–values	*β*	*P*–values
Direct and indirect significant effects^a^										
Wave 1										
Maternal caregiver age	−0.10	<0.001	−	−	−0.09	0.001	−	−	−0.06	0.025
Traumatic events	0.27	<0.001	0.09	0.002	0.12	<0.001	(0.10)	<0.001	0.06	0.018
Depressive symptoms	−	−	0.24	<0.001	0.12	<0.001	0.15	<0.001	(0.15)	0.046
Wave 2										
Child's gender	−	−	−	−	0.06	0.039	−	−	(0.02)	0.041
Depressive symptoms	−	−	−	−	0.35	<0.001	0.27	<0.001	(0.23)	<0.001
CBCL problems	−	−	−	−	−	−	0.08	<0.015	0.36	<0.001
Wave 3										
Depressive symptoms	−	−	−	−	−	−	−	−	0.33	<0.001
CBCL problems	−	−	−	−	−	−	−	−	−	−
Model summary										
*χ*²-test of model fit	9.6; d.f. = 9; *P* = 0.38									
RMSEA (90% CI)	0.008 (0.000–0.035)									
CFI	0.999									
TLI	0.999									
SRMR	0.013									

Child's gender was measured as girls (0) and boys (1). Maternal caregiver age was measured at wave 1 (in years). Traumatic events was defined as the total number of conflict-related traumatic events. Depressive symptoms at waves 1–3 were measured by the Edinburgh Postnatal Depression Scale (total score continuous). Total CBCL problems score was measured at waves 2 and 3 (total score continuous). All the variables included in the models are based on observed data and no latent variables are used. CBCL, Child Behavior Checklist; RMSEA, root-mean-square error of approximation; CFI, comparative fit index; TLI, Tucker–Lewis Index; SRMR, standardised root-mean-square residual.

a. Indirect effects are presented inside parentheses.

#### Associations between maternal depressive symptom score and child mental health

At each wave, a maternal depressive symptom score suggestive of depression was associated with poorer child mental health (cross-sectional association at wave 2: *β* = 0.35, *P* < 0.001; cross-sectional association at wave 3: *β* = 0.33, *P* < 0.001). Time-lagged associations showed the same pattern. The maternal depressive symptom score at wave 1 was associated with child mental health at wave 2 (*β* = 0.12, *P* < 0.001), and the maternal depressive symptom score at wave 2 showed an indirect association with child mental health at wave 3 (indirect standardised coefficient 0.23, *P* < 0.001) (Fig. 1).

A reciprocal effect was also evident in that there was a time-lagged relationship between child mental health at wave 2 and maternal depressive score at wave 3 (*β* = 0.08, *P* = 0.02) (Fig. 1 and Table 3).

#### Association between conflict-related traumatic events, maternal depressive scores and child mental health across waves

Past conflict-related traumatic events (measured at baseline only) were significantly associated with maternal depressive symptoms at wave 1 (*β* = 0.27, *P* < 0.001) and wave 2 (*β* = 0.09, *P* < 0.01), showing an indirect relationship with the maternal depressive score at wave 3 (indirect standardised coefficient 0.10, *P* < 0.001). In addition, past conflict-related traumatic events had a direct association with child mental health both at wave 2 (*β* = 0.12, *P* < 0.001) and wave 3 (*β* = 0.06, *P* = 0.02) (Fig. 1).

## Discussion

We conducted a robust longitudinal study of maternal depressive symptoms and mental health in early childhood in a low-resource, conflict-affected setting. We have shown that maternal depressive symptoms and child psychological problems are sustained but decreased in severity over time, and that maternal depressive symptoms are longitudinally associated with child mental health. Maternal depressive symptom scores at wave 1 were associated with poor child mental health at wave 2. It was notable that maternal depressive symptom scores at wave 2 also showed an indirect association with child mental health problems at wave 3, indicating a reciprocal time-lagged relationship between child mental health at wave 2 and maternal depressive score at wave 3.

The prevalence of depression and strength of the relationship with child mental health problems underscores the importance of targeting maternal mental health, particularly antenatal depression, in routine antenatal care in these settings. Studies have shown that in low- and middle-income countries, women are generally seen by primary healthcare workers, who may have limited training and resources in the recognition and treatment of depression, and little awareness of the serious nature of maternal depression and its potential effects on child mental health.^13^ There is also a critical need for more local health employees in non-specialised health and social care systems to be able to recognise symptoms of depression in conflict-affected populations. Psychosocial interventions have been used successfully in low- and middle-income countries, and these need to be designed, tested and funded in conflict-affected settings. Trauma-related factors need to be included in interventions for maternal depression, to acknowledge and address social and economic adversities that commonly affect trauma-affected populations who have been exposed to war and conflict.^16^

The finding that child psychological problems in turn may exacerbate maternal depressive symptoms suggests that future research in the field should consider this trajectory, which challenges an orthodoxy assuming maternal mental disorder leads to problematic child mental health, usually by way of maternal trauma and impaired parenting.^13^ More studies are required to examine the effects of the daily burden of managing challenging child psychosocial concerns on the development or exacerbation of depression in maternal caregivers. Practitioners need to be aware of the importance of supporting caregivers to manage challenging behaviours in children and in parenting under situations of extreme stress, rather than to merely treat the caregiver presenting with a mental illness.

It is important to note that younger caregivers reported worse child mental health at both waves 2 and 3, indicating that maternal depression and young age may be a combined indicator of risk for psychosocial problems in children. It is highly probable that younger women with depression have more challenges with parenting, and that, as the literature suggests, parenting in that context is a precursor for impaired child mental health. Younger maternal caregivers from conflict-affected settings also manage additional pressures arising from trauma-related stressors, such as negotiating relationships with trauma-affected male partners and intimate partner violence.^17^

Our study reinforces the needs for children in the care of conflict-affected maternal caregivers with depressive symptoms to be monitored and supported, to ensure optimal development and mental health. Our child mental health scores were higher for boys than for girls at waves 2 and 3. This is a unique finding that requires further study. At the clinical level, early childhood and maternal healthcare workers need to be aware that boys and girls of maternal caregivers with depression may be seen to have different behavioural problems by their mothers, according to their gender.

Past conflict-related traumatic events were found to have an effect on maternal depressive symptoms, the association between depression symptoms and child mental health, and child mental health directly. Traumatic events were found to be significantly associated with the maternal depressive symptom scores at waves 1 and 2, and there was an indirect relationship with the maternal depressive score at wave 3. We found that past conflict-related traumatic events in the maternal caregiver also had a direct association with child mental health at wave 2 and wave 3. These findings underscore the need for trauma-informed care to be integrated into training of health workers in conflict-affected countries. Trauma-informed care includes screening for traumatic events in routine antenatal care, front-line workers being skilled and confident enough to inquire into caregiver's past exposure to conflict-related traumas, and pragmatic interventions that acknowledge and address past violations and losses.^22^ The finding also demonstrates why political effort must be directed to prevent ongoing conflict in countries that are fragile and already affected by war.

Our study shows the importance of recognising multiple forms of stress related to war or conflict trauma, as well as parenting children with psychosocial challenges, on women's risk for depression. More practical help with parenting and mental health support, applying an integrated and holistic approach, is appropriate in refugee and conflict settings for women of childbearing age.

Relative to other longitudinal studies in the conflict-affected field, this is a very large sample of maternal caregivers and their children, enabling us to identify whether child exposure to maternal depressive disorder is associated with child mental health. In addition, we were able to examine a range of maternal trauma-related factors on child mental health. A major strength of this study is the longitudinal study design, which is rare in this field. We were not limited by a cross-sectional design yielding contemporaneous correlational data only. This allowed for the detailed examination of directionality in relationships and mediating factors. The statistical analysis using structural equation modelling with a path model were additional strengths. Another strength of the study is the measures, which are widely used in the field, and have been rigorously tested across cultures to enable comparisons with other studies. The measures are also undertaken face to face, with research assistants who spoke the same language as the participants.

Limitations include parental self-reports of depression using the EPDS, which is a screen rather than a diagnostic measure, and is known to be highly sensitive, leading to an overestimate of the prevalence of clinical depression. Also, it is important to note that women who are depressed may overreport negative behaviours of children. We did not measure disturbed parenting behaviour, which is therefore only theoretically assumed to be an important mechanism linking maternal depression and child mental health outcomes. Fathers were not included in this study, and we did not examine the effects of the spousal relationship or intimate partner violence on maternal depression or child mental health.

Our local Timorese staff are skilled in administering the culturally tested measures used in this study in the *lingua franca* Tetum, and they have had extensive training and supervisor monitoring to ensure rater reliability. Given the low levels of numeracy and literacy in Timor-Leste, however, we cannot assume that every participant fully comprehended the intended meaning of each question. Of note, however, is the internal reliability (Cronbach's alpha) found in this study for the main measures, including depressive symptoms and the CBCL. For depressive symptoms at waves 1, 2 and 3, the findings of 0.70, 0.79, and 0.80, respectively, were statistically reliable and acceptable (Cronbach's alpha 0.70–0.80). Similarly, internal reliability of the reported data for the CBCL items at waves 2 and 3 were high, at 0.92 and 0.91, respectively.

During the longitudinal period (from wave 1 to wave 3) about 33% of the wave 1 participants were lost to follow-up. Women who were lost to follow-up were comparatively younger than those retained in the sample (mean age at baseline: 25.3 *v*. 26.4 years). The differences in reported levels of education at baseline were not statistically significant between the two groups (*P* = 0.064); however, comparatively more women from the higher education group were lost to follow-up (21.3 *v*. 19.5%; *P* = 0.402). The prevalence of any intimate partner violence at baseline did not differ significantly between the two groups (58.9 *v*. 55.0%; *P* = 0.125). The differences in prevalence of maternal depression at baseline was not found to be statistically significant between the two groups (21.6 *v*. 18.7; *P* = 0.159); a similar non-significant difference was also observed in baseline mean depression score (8.6 *v*. 8.5; *P* = 0.697). Because of the large number women retained in the wave 3 sample (*n* = 1118), the loss of 33% of participants in the context of the reported differences can be assumed to have occurred randomly, and therefore the loss to follow-up should not affect the overall findings of this study unduly. We need to be cautious when interpreting our results concerning women under 25 years of age across three waves, given that over the follow-up period, a proportion of women from this younger age group have naturally moved to older age groups.

Despite these limitations, our study was able to identify important pathways in the relationship between maternal depressive symptoms and early child mental health problems in an understudied, conflict-affected and displaced population. These findings are novel, and indicate that parenting skills programmes, particularly targeting younger parents, should be trialled in conflict-affected settings. Furthermore, our study demonstrates the importance of a trauma-informed approach to maternity care, including the importance of screening for depression and traumatic events that may affect maternal mental health.

## Data Availability

The data that support the findings of this study are available from the corresponding author, S.J.R., upon reasonable request.

## Supplementary material

For supplementary material accompanying this paper visit http://doi.org/10.1192/bjo.2022.20. click here to view supplementary material
