# Supplementary material for: The impact of maternal depressive symptoms and traumatic events on early childhood mental health in conflict-affected Timor-Leste
Source: BJPsych Open. 2022 Feb 24;8(2):e51. doi: 10.1192/bjo.2022.20 (PMC8935917; doi:10.1192/bjo.2022.20)
Supplement: Supplementary file 1 [file S2056472422000205sup001.docx]

**Participant flow chart covering Wave 1, Wave 2, and Wave 3 survey**

**Not interviewed: 68**

**66** could not be contacted

**2** refused to participate

**279** Could not be traced

**5** Died

**8** Too sick or mentally incapable for interview

(total 292 of wave 1 missed out at wave 2)

**Wave 1 survey**

**1672** interviewed

**96%** response rate (1672 out of 1740)

**Wave 2 survey**

**1380** of the Wave 1 cohort of 1672 identified for interview

**Wave 2 survey**

**1303** interviewed

**78%** retention rate (1303 out of 1672)

**94%** response rate (1303 out of 1380)

**March 2014 and November 2016**

**Wave 3 survey**

**1221** of the of the Wave 1 cohort identified for interview at Wave 3 (out of 1303)

**Not interviewed: 77**

**69** could not be contacted

**8** refused to participate

**Wave 1 survey**

**1740** pregnant women eligible for interview

**Wave 3 survey**

**1140** interviewed at Wave 3

**68.2%** retention rate (1140 out of 1672)

**93.4%** response rate (1140 out of 1221)

**March 2014 and November 2016**

**Not interviewed: 81**

**79** could not be contacted

**2** refused to participate

22 excluded due to incomplete or missing data

**1118** included in the analysis

**March 2014 and November 2016**

**80** Could not be traced

**2** Died

(82 of wave 2 missed out at wave 3)
